# Anti-TNFα Treatment Impairs Long-Term Immune Responses to COVID-19 mRNA Vaccine in Patients with Inflammatory Bowel Diseases

**DOI:** 10.3390/vaccines10081186

**Published:** 2022-07-26

**Authors:** Keren Masha Rabinowitz, Michal Navon, Hadar Edelman-Klapper, Eran Zittan, Ariella Bar-Gil Shitrit, Idan Goren, Irit Avni-Biron, Jacob E. Ollech, Lev Lichtenstein, Hagar Banai-Eran, Henit Yanai, Yifat Snir, Maor H. Pauker, Adi Friedenberg, Adva Levy-Barda, Arie Segal, Yelena Broitman, Eran Maoz, Baruch Ovadia, Maya Aharoni Golan, Eyal Shachar, Shomron Ben-Horin, Nitsan Maharshak, Michal Mor, Haim Ben Zvi, Rami Eliakim, Revital Barkan, Tali Sharar-Fischler, Sophy Goren, Noy Krugliak, Edward Pichinuk, Michael Mor, Michal Werbner, Joel Alter, Hanan Abu-Taha, Kawsar Kaboub, Moshe Dessau, Meital Gal-Tanamy, Dani Cohen, Natalia T. Freund, Iris Dotan, on behalf of the Responses to COVID-19 Vaccine Israeli IBD Group

**Affiliations:** 1 Division of Gastroenterology, Rabin Medical Center, Petah Tikva 4941492, Israel; k.rabinowitz@gmail.com (K.M.R.); hadared@clalit.org.il (H.E.-K.); idangoren@gmail.com (I.G.); iritab@clalit.org.il (I.A.-B.); jacobel@clalit.org.il (J.E.O.); hagarb@clalit.org.il (H.B.-E.); henityanai@gmail.com (H.Y.); yifats3@clalit.org.il (Y.S.); maorha11@clalit.org.il (M.H.P.); adifr2@clalit.org.il (A.F.); broytman@clalit.org.il (Y.B.); mayago1@clalit.org.il (M.A.G.); revitalba11@clalit.org.il (R.B.); talish43@clalit.org.il (T.S.-F.); hananabutaha@mail.tau.ac.il (H.A.-T.); kawsarkaboub@mail.tau.ac.il (K.K.); 2 Felsenstein Medical Research Center, Sackler School of Medicine, Tel Aviv 69978, Israel; 3 Department of Clinical Microbiology and Immunology, Sackler Faculty of Medicine, Tel Aviv University, Tel Aviv 69978, Israel; michalnavon@mail.tau.ac.il (M.N.); noykrugliak@mail.tau.ac.il (N.K.); mordekovich@mail.tau.ac.il (M.M.); 4 Sackler Faculty of Medicine, Tel Aviv University, Tel Aviv 69978, Israel; eyal.shahar@sheba.health.gov.il (E.S.); shomron.ben-horin@sheba.health.gov.il (S.B.-H.); nitsanm@tlvmc.gov.il (N.M.); haimbe@clalit.org.il (H.B.Z.); abraham.eliakim@sheba.health.gov.il (R.E.); 5 The Abraham and Sonia Rochlin IBD Unit, Department of Gastroenterology, Emek Medical Center, Afula 1834111, Israel; eranzittan@gmail.com; 6 Technion-Israel Institute of Technology, Rappaport Faculty of Medicine, Haifa 3109601, Israel; 7 Shaare Zedek Medical Center, Digestive Diseases Institute, Jerusalem 9103102, Israel; ariellash@szmc.org.il; 8 Faculty of Medicine, Hebrew University of Jerusalem, Jerusalem 9112102, Israel; 9 Clalit Health Services, Petah Tikva 4933355, Israel; levli@clalit.org.il; 10 The Adelson School of Medicine, Ariel University, Ariel 4077625, Israel; 11 Biobank, Rabin Medical Center, Department of Pathology, Petah Tikva 4941492, Israel; advale11@clalit.org.il; 12 The Institute of Gastroenterology and Hepatology, Soroka University Medical Center, Beer-Sheva 84101, Israel; arikse@clalit.org.il; 13 Clalit Health Services, Tel Aviv 4940612, Israel; eranma@clalit.org.il (E.M.); michalmo2@clalit.org.il (M.M.); 14 Department of Gastroenterology and Hepatology, Hillel Yaffe Medical Center, Hadera 38100, Israel; ovadiabaruch@gmail.com; 15 Department of Gastroenterology, Sheba Medical Center, Ramat Gan 52621, Israel; 16 Department of Gastroenterology and Liver Diseases, Tel Aviv Medical Center, Tel Aviv 64239, Israel; 17 Microbiology Laboratory, Rabin Medical Center, Petah Tikva 4941492, Israel; 18 Sackler Faculty of Medicine, School of Public Health, Tel Aviv University, Tel Aviv 69978, Israel; sophyg@tauex.tau.ac.il (S.G.); dancohen@tauex.tau.ac.il (D.C.); 19 Blavatnik Center for Drug Discovery, Tel Aviv University, Tel Aviv 69978, Israel; pichinuk@tauex.tau.ac.il; 20 Molecular Virology Laboratory, The Azrieli Faculty of Medicine, Bar-Ilan University, Safed 1311502, Israel; michal.poran@biu.ac.il (M.W.); mgtanamy@gmail.com (M.G.-T.); 21 The Laboratory of Structural Biology of Infectious Diseases, The Azrieli Faculty of Medicine, Bar-Ilan University, Safed 1311502, Israel; yoel.alter@biu.ac.il (J.A.); moshe.dessau@biu.ac.il (M.D.)

**Keywords:** COVID-19, vaccine, mRNA-BNT162b2, anti-SARS-CoV-2 antibodies, serologic response longevity, circulating B cells, cross-reactivity

## Abstract

Patients with inflammatory bowel disease (IBD) treated with anti-tumor-necrosis factor-alpha (TNFα) exhibited lower serologic responses one-month following the second dose of the COVID-19 BNT162b2 vaccine compared to those not treated with anti-TNFα (non-anti-TNFα) or to healthy controls (HCs). We comprehensively analyzed long-term humoral responses, including anti-spike (S) antibodies, serum inhibition, neutralization, cross-reactivity and circulating B cell six months post BNT162b2, in patients with IBD stratified by therapy compared to HCs. Subjects enrolled in a prospective, controlled, multi-center Israeli study received two BNT162b2 doses. Anti-S levels, functional activity, specific B cells, antigen cross-reactivity, anti-nucleocapsid levels, adverse events and IBD disease score were detected longitudinally. In total, 240 subjects, 151 with IBD (94 not treated with anti-TNFα and 57 treated with anti-TNFα) and 89 HCs participated. Six months after vaccination, patients with IBD treated with anti-TNFα had significantly impaired BNT162b2 responses, specifically, more seronegativity, decreased specific circulating B cells and cross-reactivity compared to patients untreated with anti-TNFα. Importantly, all seronegative subjects were patients with IBD; of those, >90% were treated with anti-TNFα. Finally, IBD activity was unaffected by BNT162b2. Altogether these data support the earlier booster dose administration in these patients.

## 1. Introduction

Patients with inflammatory bowel disease (IBD), including Crohn’s disease (CD) and ulcerative colitis (UC), are often treated with immunomodulators and/or biologic therapy such as anti-tumor necrosis factor alpha (TNFα), potentially associated with an increased risk of infection [1,2,3,4]. Their ability to mount an adequate immune response to infections or following vaccination is limited [5,6]. This is specifically concerning during the severe acute respiratory syndrome coronavirus 2 (SARS-CoV-2) pandemic, which led to the use of new vaccines [7,8]. Lower SARS-CoV-2 vaccine responses were reported in immunocompromised patients [9,10], as well as in patients with IBD treated with certain therapies, specifically anti-TNFα [11,12,13]. However, data regarding the durability of protection, susceptibility to breakthrough infections as well as long-term side effects are scarce. Furthermore, it was recently shown that vaccinated immunosuppressed individuals who mount limited anti-SARS-CoV-2 immune responses contribute to the evolution of new viral strains [14]. Therefore, it is crucial to determine the magnitude of protection in vaccinated patients with IBD, specifically those treated with anti-TNFα overtime and against emerging variants of concern (VOCs).

Recently, we and others demonstrated that patients with IBD treated with anti-TNFα had a significantly lower serologic response observed already 4 weeks after two doses of the mRNA-based BNT162b2 vaccine [11,12,15]. In the current study, we aimed to continue the prospective assessment of long-term immune responses in patients with IBD stratified according to therapy, focusing on the differential response one and six months post BNT162b2. Specifically, we investigated their ability to preserve long-lasting antibody and B cell responses, as well as to develop antibodies that cross-react with three emerging VOCs six months following vaccination. We demonstrated that in patients with IBD treated with anti-TNFα, compared to anti-TNFα untreated and healthy control groups, a dramatic reduction in antibody longevity, cross-reactivity and immune memory was observed.

## 2. Materials and Methods

### 2.1. Study Design and Participants

We conducted a prospective, observational, multi-center study to assess short- and long-term immune responses to BNT162b2, their dynamics, predictors of response and safety in a cohort of patients with IBD compared to healthy controls (HCs). The short-term part of this study was previously described in ref [12]. Briefly, patients ≥18 years were recruited. IBD diagnosis was defined by accepted criteria. The HC group included volunteers (healthcare professionals and their relatives) without known gastrointestinal diseases. Patients with IBD were stratified at baseline into those treated with anti-TNFα or those with any other IBD treatment or no medical treatment. All participants received two 30 µg BNT162b2 vaccine doses intra-muscularly, administered 21–28 days apart, as per manufacturers’ recommendations. The study was approved by the local IRBs at the Rabin, Shaare Zedek, Emek and Soroka Medical Centers, (1072-20-RMC, 0557-20-SZMC, 0247-20-EMC and 0568-20-SOR, respectively). MOH number: 2020-12-30_009617. All participants signed an informed consent form before any study procedure.

### 2.2. Study Procedure

**Figure 1:**
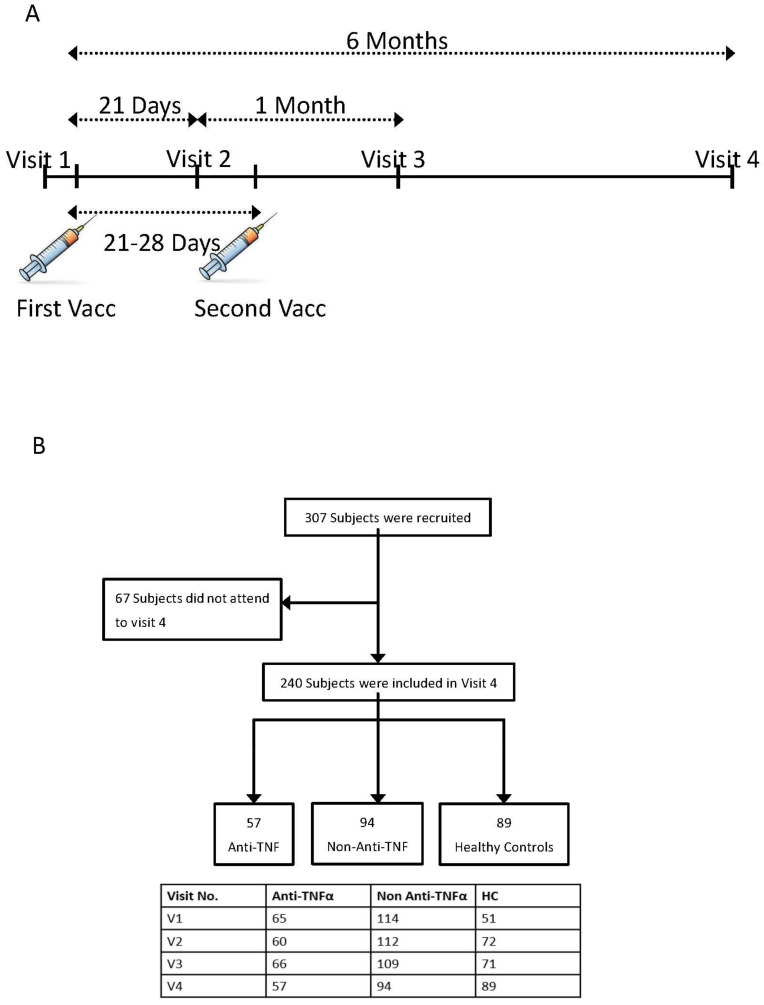
(**A**) Study protocol. Patients were enrolled at visit 1, before the first vaccine dose. Visit 2 was 14–21 days after the first but before the second vaccine dose. Visit 3 and 4 were one and 6 months after the first vaccine dose, respectively. In each visit, laboratory tests were performed and questionnaires regarding disease severity and adverse events (AEs) were filled. (**B**) Patient disposition. The diagram represents all enrolled participants who were recruited before vaccination. In total, 25 subjects were recruited at the second visit (after first vaccine dose but before the second one), mainly due to logistic reasons. Most of them (19) were healthy controls (HCs). Number of subjects at each visit is detailed in the table below the diagram. Abbreviations: HC—healthy control; Vacc—vaccine dose.

Eligible participants were evaluated at 5 time points: (i) before the first vaccine dose—visit 1; (ii) 14–21 days after the first and before the second vaccine dose—visit 2; (iii) phone call a week after the second vaccine dose to report adverse events (AEs); (iv) 21–35 days after the second vaccine dose—visit 3; (v) 6 months after the first dose—visit 4 (see Figure 1A). At enrolment, patients were assessed for baseline demographic and IBD characteristics. Specifically, medical treatment, duration and dose were registered. At each visit, a clinical evaluation was performed using IBD-specific questionnaires—the Harvey–Bradshaw index (HBI) [16] and the simple clinical colitis activity index (SCCAI) [17].

Laboratory tests were performed at all visits, including a complete blood count, C-reactive protein (CRP), COVID-19 serology and functional neutralization and inhibition assays. Serum was separated from collected blood, aliquoted and stored at −80 °C until further analyses. At visits 3 and 4, 3–4 patients in each experimental group donated an additional EDTA whole-blood tube for PBMCs isolation. PBMCs were stored in liquid nitrogen until further analysis.

### 2.3. Laboratory Methods

SARS-CoV-2 anti-S IgG II quantitative testing was performed using the Abbott architect i2000sr platform following the manufacturer’s instructions [18]. Anti-S values ≥ 50 activity units (AU)/mL were considered positive.

SARS-CoV-2 nucleocapsid (N) IgG testing was performed semi-quantitatively using ELISA plates coated with N protein following the manufacturer’s instructions (EUROIMMUN, Lubeck, Germany). Values ≥ 1.1 units were considered positive. Anti-N was assessed in all subjects at visits 3 and 4. For all those testing positive, the existence of anti-N antibodies was assessed at all the other visits.

Methods of lymphocyte analytical determination. Peripheral blood samples were collected in K3-EDTA anticoagulant and processed within 3 h of collection. Blood cell counts were performed on an automated hematology analyzer (Advia 2120i). Anticoagulated venous blood was aliquoted in 12.75 mm polypropylene tubes (Beckman coulter) and incubated in the dark for 15 min at room temperature with the appropriate fluorochrome-conjugated monoclonal 4–6 antibody combinations at the manufacturer’s recommended concentration (Beckman Coulter and Dako). Fluorescence analysis was performed using a Beckman–Coulter Navios multiparameter flow cytometer and later analyzed with Kaluza or Navios Software. A minimum of 30,000 events was collected. Absolute counts of circulating cell subsets were calculated using the percentages obtained with flow cytometry, and the leukocyte count obtained with the hematological analyzer.

Receptor-binding domain (RBD): angiotensin-converting enzyme (ACE) 2 inhibition ELISA was performed as previously described by [10,12,19] incubating a mix of serum and RBD from Wuhan-1 strain with ACE2-coated plates (ACE2 was produced in house). Inhibition percentage was calculated for each well with the following formula: $\left(1-\frac{\left[\mathrm{RBD}-\mathrm{serum}\;O.D.\right]}{\left[\mathrm{only}\;\mathrm{RBD}\;O.D.\right]}\right)\times 100$. Negative results, indicating no inhibition, were set as 0% inhibition.

Preparation of SARS-CoV-2 spike pseudo-particles and neutralization assay was performed as previously described [12].

Flow cytometry anti-RBD BCR staining. PBMCs were first thawed at 37 °C and then washed with 14 mL RPMI 1640 medium. Cells were resuspended in FACS buffer (1% FBS in PBS ×1 and 2 mM EDTA) and stained with anti-CD19-PerCP-Vio 700 (Miltenyi Biotec, Bergisch Gladbach, Germany, 130-113-733), anti-IgG-FITC (Miltenyi Biotec, Bergisch Gladbach, Germany, 130-118-479) and anti-IgA-VioBlue (Miltenyi Biotec, Bergisch Gladbach, Germany, 130-113-479) and labeled biotinylated RBD via streptavidin-APC (Miltenyi Biotec, 130-106-792) and streptavidin-PE (Miltenyi Biotec, Bergisch Gladbach, Germany, 130-106-790). Cells were recorded on Cytoflex L4 instrument (Beckman Coulter, Brea, CA, USA) and analyzed via FlowJo software version 10 (BD, Franklin Lakes, NJ, USA). Serologic response against VOCs RBD was performed in high-binding 384-well ELISA plates (Greiner Bio-One, Kremsmünster, Austria, 781061). Plates were coated with 12.5 µL of 0.5 µg/mL RBD corresponding to the different VOCs in PBS ×1, and incubated overnight at 4 °C. The following day, the coating was discarded, plates were washed twice with PBS ×1 with 0.05% Tween20 (Sigma-Aldrich, St. Louis, MI, USA) (“washing buffer”) and blocked for 2 h at room temperature with 80 µL PBS ×1, 3% BSA (MP Biomedicals, Irvine, CA, USA) 20 mM EDTA and 0.05% Tween20 (“blocking buffer”). Plasma samples were diluted 1:250 in blocking buffer and incubated for 1 h at room temperature, followed by 3 washes with washing buffer. Secondary anti-IgG antibody conjugated to horseradish peroxidase (Jackson ImmunoResearch, Cambridge, UK, 109-035-088) was diluted to 1:5000 in blocking buffer, added to each well and incubated for 45 min at room temperature. Following four additional washes with wash buffer, 30 µL of TMB/E (abcam, Cambridge, UK, ab171523) was added to each well and the absorbance was read after 20 min at 650 nm (Tecan, Männedorf, Switzerland, SPARK).

### 2.4. Statistical Analysis

Study data were collected and managed using REDCap electronic data capture tools hosted at Clalit health services [20,21]. REDCap (Research Electronic Data Capture) is a secure, web-based software platform designed to support data capture for research studies. Data was analyzed using SPSS version 28 (IBM, New York, NY, USA). All tests were two-tailed and *p* < 0.05 was considered significant. Anti-S levels were expressed as geometric mean concentrations (GMCs) with 95% confidence intervals (CIs). Other continuous data were reported as median and IQR unless otherwise stated. Counts and percentages were employed for categorical variables. Univariate analyses using independent sample *t*-test, one-way analysis of variance (ANOVA) with Bonferroni multiple-comparison correction or Kruskal–Wallis non-parametric test of ln-transformed anti-S levels and Spearman’s rank correlation coefficients were used to identify demographic, disease, vaccine and treatment-related factors associated with anti-S levels. Correlations between the various immunological outcomes were also assessed. We used multi-variate stepwise linear regression models to identify factors independently associated with ln anti-S levels. Standardized beta coefficients were obtained from linear regression.

## 3. Results

### 3.1. Study Population

The cohort included 307 subjects recruited from four medical centers in Israel, analyzed before the first vaccine dose (230/307, visit one), three weeks after the first dose (244/307, visit two) and one month after second the dose (246/307, visit three). Results of this cohort’s short-term serologic response were previously reported [12]. A total of 240/307 of the initially recruited subjects was examined in the current study six months following the first dose (visit four). Of those, 151/240 were patients with IBD, and 89 subjects were healthy controls (HCs group). Within the IBD patients’ group, 57 were treated with anti-TNFα agents (anti-TNFα group), while 94 were treated with any other medical treatment or no medical treatment at all (non-anti-TNFα group) (Figure 1B). Baseline characteristics are detailed in Table 1. Patients were examined 176 (166–186) (median (IQR)) days after the first vaccine dose. Here, we compared the response to BNT162b2 between visit three and visit four.

Patients with IBD treated with anti-TNFα exhibited a steeper anti-SARS-CoV-2 antibody decay, and significantly lower neutralizing and inhibitory activity six months post vaccination.

**Figure 2:**
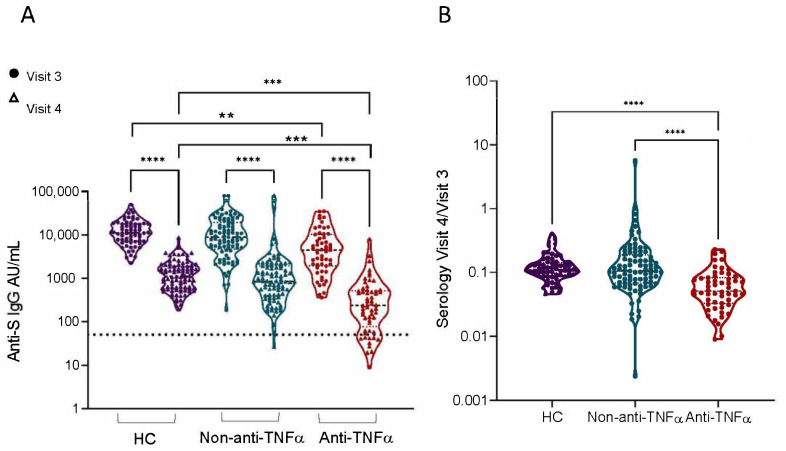
Patients with IBD treated with anti-TNFα showed significantly reduced levels of anti-S levels 6 months after two BNT162b2 vaccine doses. (**A**) Levels of anti-S levels in sera from healthy controls (HCs, shown in purple), patients with IBD receiving non-anti-TNFα treatment (non-anti-TNFα, shown in blue) and patients with IBD receiving anti-TNFα treatment (anti-TNFα, shown in red). Antibodies were measured with the Abbott quantitative anti-S IgG kit. Visits 3 (filled circles) and 4 (open triangles)—after two vaccine doses, 1 and 6 months, respectively. Statistical analysis was carried out using independent sample Kruskal–Wallis test. ****—*p* < 0.0001, ***—*p* < 0.001, **—*p* < 0.01, Black solid line denotes median, black dashed line denotes IQR25-75. Dotted line represents the threshold for seroconversion (50 AU/mL). Specific GMCs and *p*-values in Supplementary Table S1. (**B**) Ratio between visit 4 and visit 3 anti-S levels.

SARS-CoV-2 anti-S levels declined over time, both after natural infection and following vaccination [22,23,24,25]. One month after receiving the second vaccine dose (visit three) all subjects had positive anti-S levels, with anti-TNFα-treated subjects exhibiting significantly lower titers [12]. At visit four, six months after vaccination, a significant decrease in anti-S levels was observed in all groups (*p* < 0.0001, Figure 2A, GMCs values are detailed in Supplementary Table S1), and the differences in antibody levels between anti-TNFα compared to the non-anti-TNFα and HCs groups were significant (*p* = 0.0024 and *p* = 0.0004, respectively; Figure 2A). Most (94.9%) recruited subjects remained seropositive. However, all 11 seronegative subjects were patients with IBD: 10 treated with anti-TNFα, and 1 with an immunomodulator (6-mercaptopurine).

IBD patients treated with anti-TNFα demonstrated significantly decreased visit four/visit three antibody titer ratios compared to the non-anti-TNFα and HCs groups (*p* < 0.0001) (Figure 2B). This indicated that subjects from the anti-TNFα group not only developed lower SARS-CoV-2 antibody titers immediately after immunization, but also lost a larger proportion of their antibodies over the 6-month period. More importantly, anti-S levels were comparable between all treatments in the non-anti-TNFα group. Specifically in patients treated with vedolizumab (n = 22) or 5-ASA (n = 18), vaccine responses were comparable to those not receiving any medical treatment (n = 31) (Supplementary Figure S1).

As breakthrough infections were reported after mRNA vaccinations [26,27], we next asked whether subjects in our cohort were exposed to SARS-CoV-2 between visits three and four. More importantly, anti-N titers reflecting infection were positive at visit three in two subjects from each group (HC, non-anti-TNFα and anti-TNFα) [12]. At visit four, only two additional patients became positive (newly infected with SARS-Cov-2). Both were patients with IBD, one treated with adalimumab and the other untreated. Interestingly, while the serologic response in the untreated patient was high (>60,000 AU), the patient treated with adalimumab was seronegative (<50 AU).

**Figure 3:**
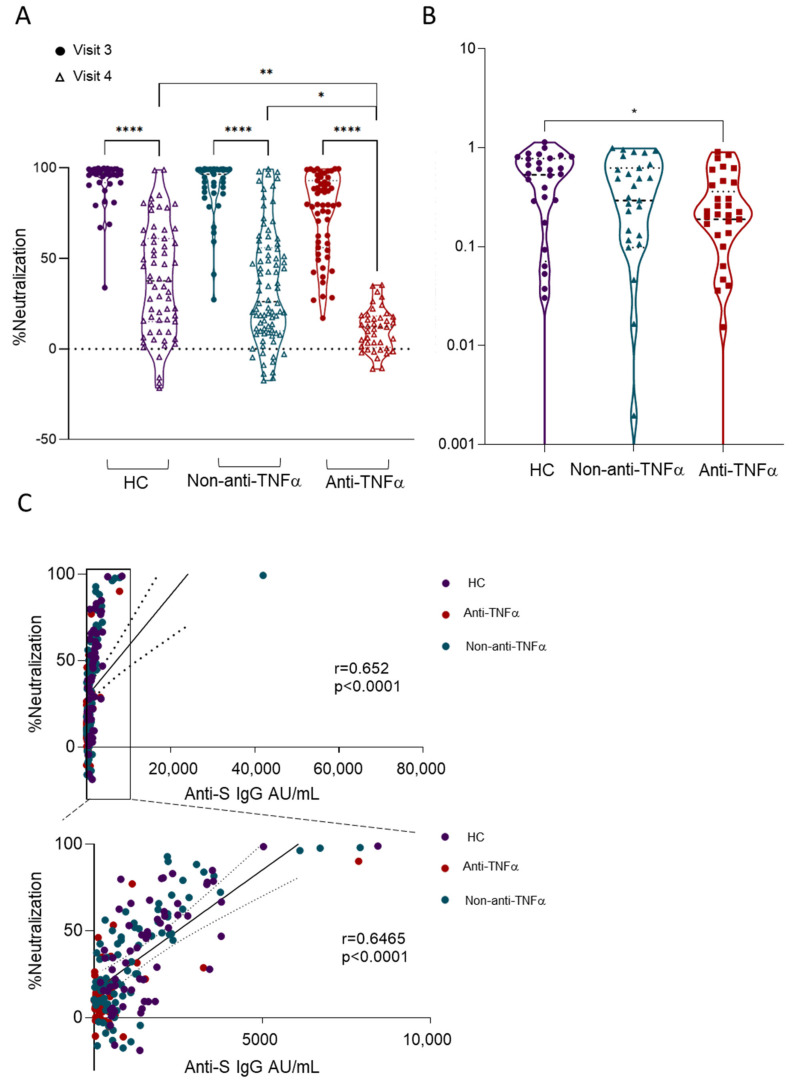
Patients with IBD treated with anti-TNFα had significantly reduced levels of anti-SARS-CoV-2-neutralizing activity 6 months after two BNT162b2 vaccine doses. (**A**) Sera, diluted to a final concentration of 1:200 from healthy controls (HCs, shown in purple), patients with IBD receiving non-anti-TNFα treatment (non-anti-TNFα, shown in blue) and patients with IBD receiving anti-TNFα treatment (anti-TNFα, shown in red) were incubated with VSV-spike pseudo-particles (VSV∆G^GFP^S∆19) for 1 h in 37 °C, prior to infecting ACE2 expressing HEK293 cells for 24 h. The number of GFP-positive cells was normalized and converted to a neutralization percentage in each sample, compared to the average of control samples. Visit 3 (filled circles), visit 4 (open triangles)—after two vaccine doses, 1 and 6 months, respectively. (**B**) Ratio between visit 4 and visit 3 anti-SARS-CoV-neutralizing activity. Statistical analysis was carried out using independent sample Kruskal–Wallis test. *—*p* < 0.05, **—*p<* 0.01, ****—*p* < 0.0001. Black solid line denotes median, black dashed line denotes IQR 25-75. (**C**) Correlations between anti-S level and neutralizing activity. Abbreviations: VSV—vesicular stomatitis virus; ACE2—angiotensin-converting enzyme-2; RBD—receptor-binding domain; HEK—human embryonic kidney.

Neutralizing antibodies are considered critical for virus control and the prevention of SARS-CoV-2 infection [28]. Therefore, we assessed serum neutralization using SARS-CoV-2 spike pseudo-particles as previously described [12] and normalized to neutralizing activity in pre-vaccination samples (visit one). Serum from all study groups six months after vaccination (visit four) had significantly reduced neutralizing activity compared to visit three (*p* < 0.0001; Figure 3A). Moreover, neutralizing activity at visit four was significantly lower in patients with IBD treated with anti-TNFα compared to both non-anti-TNFα-treated patients and HCs (*p* = 0.0077 and *p* = 0.016, respectively), and the visit four/visit three ratio was significantly lower in the anti-TNFα group compared to HCs (*p* < 0.05; Figure 3B). Neutralizing activity correlated with anti-S levels (Figure 3C), as was previously shown by us and others [12,24].

**Figure 4:**
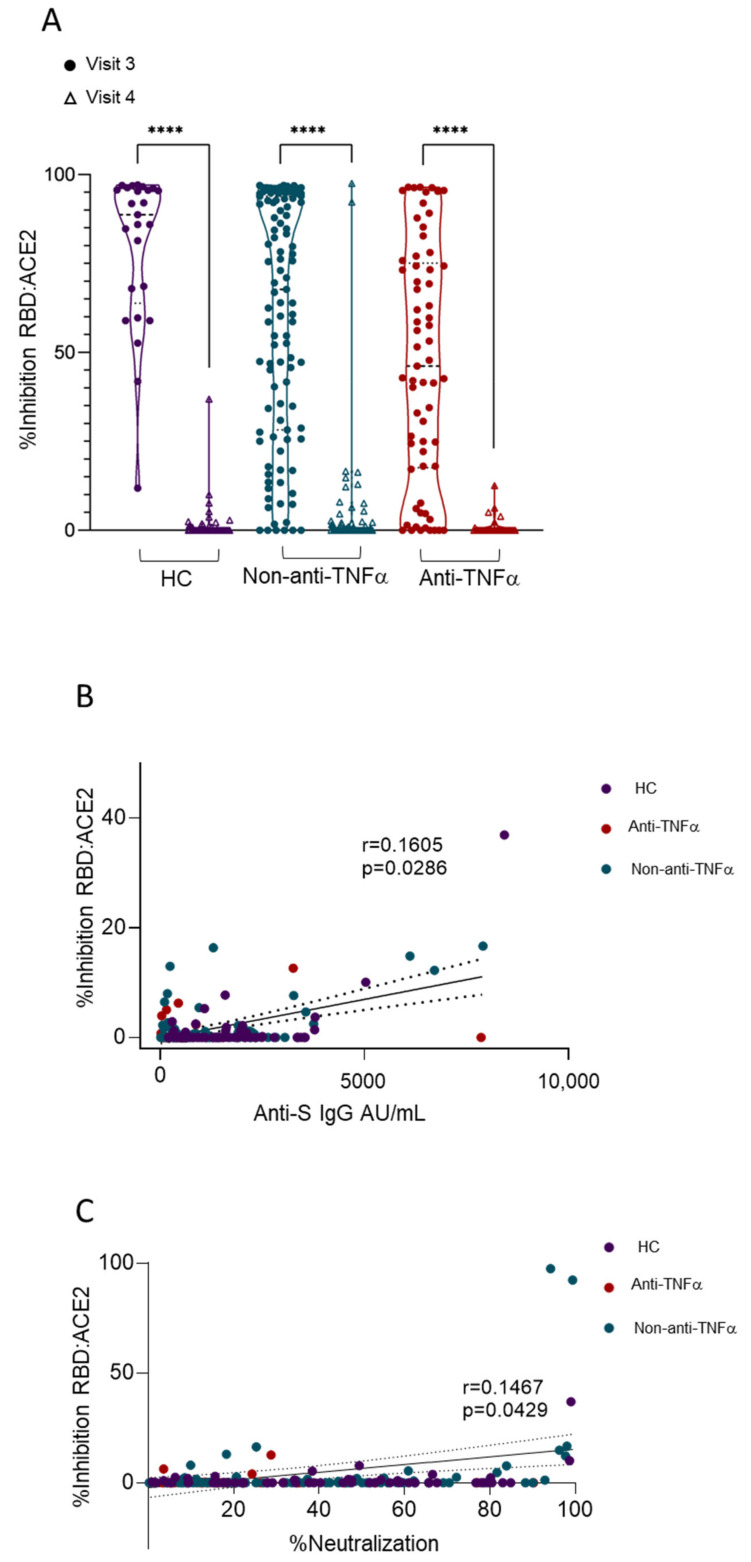
Significantly reduced levels of anti-SARS-CoV-2 inhibiting antibodies 6 months after two BNT162b2 vaccine doses in all recruited subjects. (**A**) Ability of serum from healthy controls (HCs, shown in purple), patients with IBD receiving non-anti-TNFα treatment (non-anti-TNFα, shown in blue) and patients with IBD receiving anti-TNFα treatment (anti-TNFα, shown in red) to inhibit SARS-CoV-2 RBD binding to ACE2 receptor. Values measured with ELISA and presented as % inhibition (*y* axis). Visit 3 (filled circles), visit 4 (open triangles)—after two vaccine doses, 1 and 6 months, respectively. Zero inhibition was set as the value of RBD without added sera. Statistical analysis was carried out using independent sample Kruskal–Wallis test, ****—*p* < 0.0001. (**B**) Correlation between anti-S titer and inhibition responses. (**C**) Correlation between neutralizing activity and inhibition responses. Abbreviations: RBD—receptor-binding domain; ACE2—angiotensin-converting enzyme-2.

Viral entry is mediated by the binding of SARS-CoV-2 RBD to angiotensin-converting enzyme 2 (ACE2); thus, the blocking of this interaction is a major mechanism of anti-SARS-CoV-2-neutralizing antibodies [29]. Therefore, we next assessed the ability of sera to inhibit the RBD:ACE2 interaction using competitive ELISA as previously described [10,12,19]. While, at visit three, all study groups exhibited measurable inhibitory activity, with the anti-TNFα group having the lowest compared to non-anti-TNFα-treated patients and HCs [12], at visit four, a significant reduction in inhibition activity was apparent in all subjects, regardless of the treatment regimen or disease status (Figure 4A). Moreover, the positive correlation between anti-S levels or neutralizing and RBD:ACE2 inhibitory activity at visit four were not as strong as at visit three (Figure 4B,C). The observation that both anti-S levels and neutralizing activity were still detectable at visit four, yet the inhibition of RBD:ACE2 was almost completely abolished, suggests that neutralizing activity at visit four was mediated (at least partially) by the non-RBD:ACE2 blockade, or, alternatively, that the anti-RBD titers were below the detection level of the RBD:ACE2 inhibition assay.

### 3.2. RBD-Specific Circulating B Cells Are Reduced in Patients with IBD Treated with Anti-TNFα

**Figure 5:**
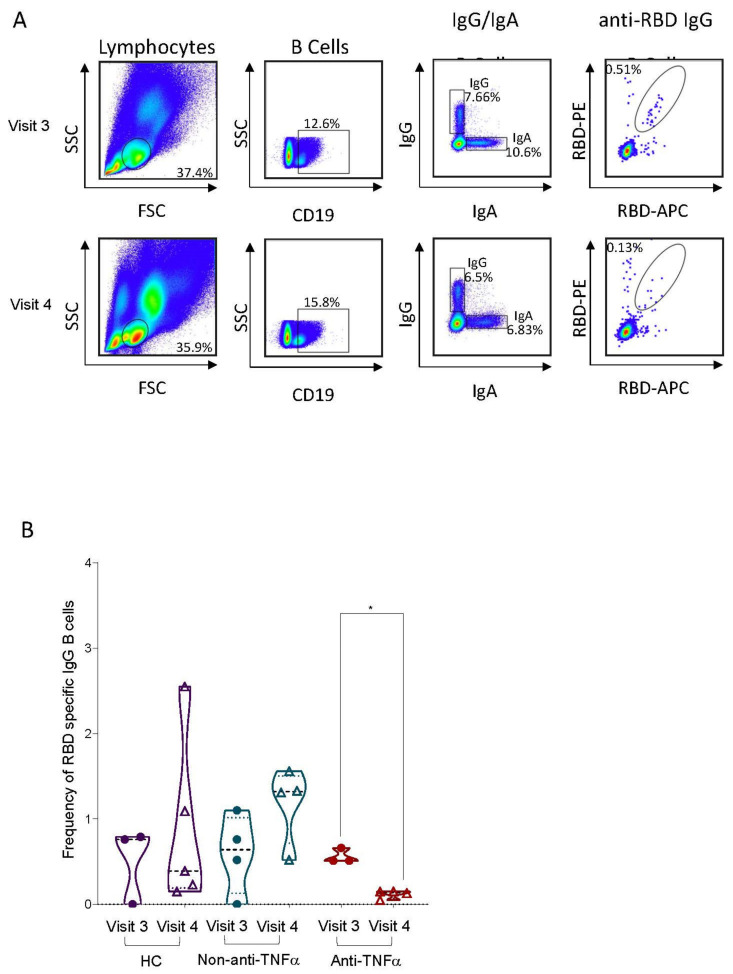
Significant reduction in peripheral RBD-specific IgG B cells over time in patients with IBD treated with anti-TNFα. (**A**) A representative gating strategy and population prevalence from flow cytometry assay at 1 and 6 months post vaccination (visit 3 and 4, respectively). (**B**) Frequency of RBD-specific IgG B cells out of peripheral IgG B cells from healthy controls (HC, shown in purple), patients with IBD receiving non-anti-TNFα treatment (non-anti-TNFα, shown in blue) and patients with IBD receiving anti-TNFα treatment (anti-TNFα, shown in red) following vaccination. Each column was separated into two columns, visit 3 and visit 4, PBMC samples from 1 and 6 months after second vaccine dose, respectively. Statistical analysis was carried out using paired mixed-effect ANOVA to compare frequencies over time for each group (*—*p* < 0.05).

While antibodies represent the immediate correlates of protection, circulating B cells are part of the long-term immunological memory, reducing disease severity in cases of re-infection and breakthrough infection, and are thought to be responsible for protection from emerging viral strains [30,31]. Moreover, it has been shown that in contrast to serum antibody titers, following the SARS-CoV-2 vaccine, the memory B cell compartment remains stable over time, and even expands over the course of six months following vaccination [23]. Therefore, we next estimated the levels of RBD-specific circulating B cells by analyzing PBMCs isolated from whole blood of three representative subjects from each of the three study groups at visit three and visit four time points (characteristics of patients donating PBMCs are detailed in Supplementary Table S2). Cells were stained for CD19, IgG and IgA, as well as for fluorophore-conjugated RBD in two different colors to detect RBD-specific circulating B cells (Figure 5A). Comparable levels of RBD-specific B cells were detected at visit three in all the groups. These levels remained stable at visit four in HCs and patients with IBD not treated with anti-TNFα. In contrast, a significant reduction in RBD-specific B cells was observed in patients with IBD treated with anti-TNFα (*p* = 0.0156 compared to visit three), to the point that the RBD-specific B cell populations were barely detectable in the three subjects analyzed (Figure 5B). We concluded that the anti-TNFα treatment in IBD patients had a profound effect on RBD-specific B cells following vaccination. Notably, total immunoglobulin levels as well as lymphocyte sub-population percentages were comparable between the groups and remained stable throughout all visits (Supplementary Figure S2).

### 3.3. Patients with IBD Treated with Anti-TNFα Exhibit Decreased Reactivity to Beta VOCs

**Figure 6:**
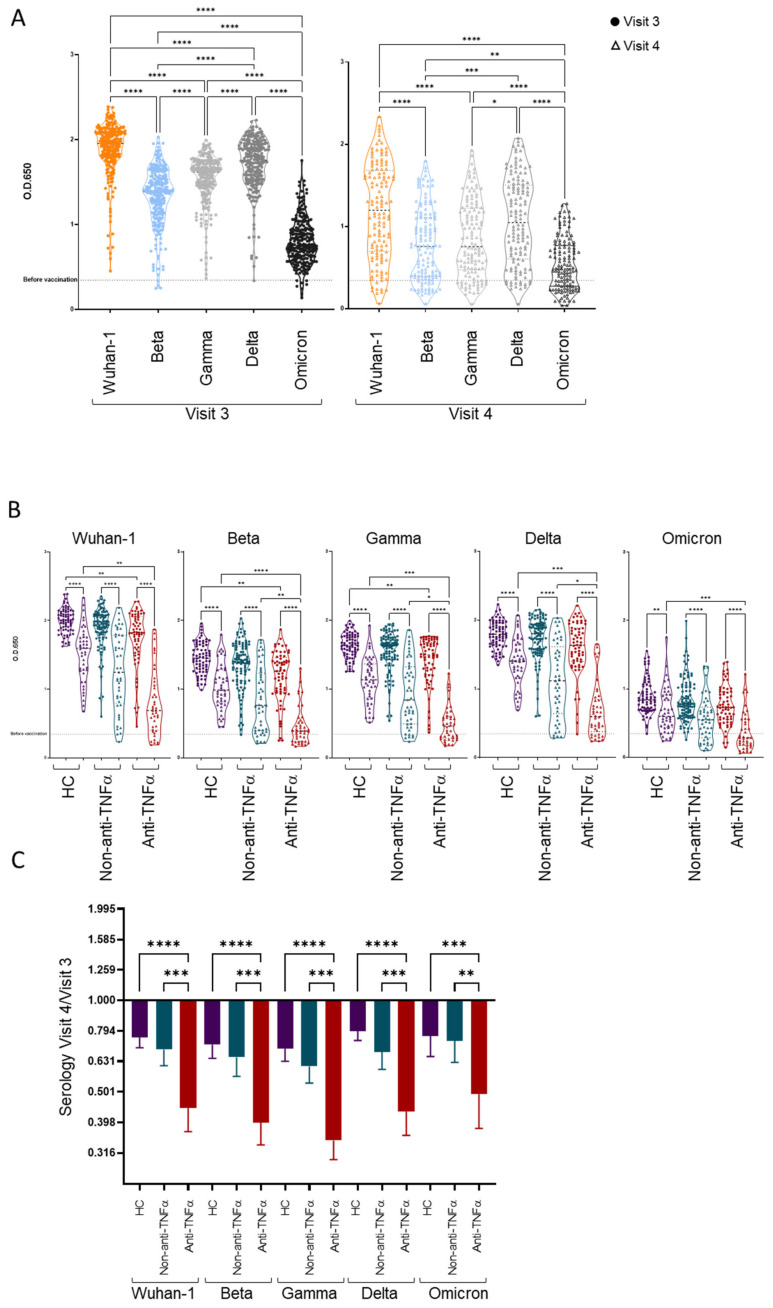
Patients with IBD treated with anti-TNFα had reduced cross-reactivity. (**A**) Ability of vaccinee sera to bind SARS-CoV-2 RBD from Wuhan-1 strain and variants of concern (VOCs)—beta, gamma, delta and omicron (Wuhan-1 in orange, beta in light blue, gamma in light grey, delta in dark grey and omicron in black)—as measured with ELISA, at two time points: 1 month post vaccination (visit 3, filled circles) and 6 month post vaccination (visit 4, open triangles). (**B**) Ability of vaccinee sera to bind SARS-CoV-2 RBD from Wuhan-1 strain and VOCs, separated to healthy controls (HCs, shown in purple), patients with IBD receiving non-TNFα treatment (non-anti-TNFα, shown in blue) and patients with IBD receiving anti-TNFα treatment (anti-TNFα, shown in red). Dotted line indicating mean O.D. value from 5 sera samples before vaccination. (**C**) Bar plot indicates mean and 95% CIs of fold change in sera binding ability over time for HCs, non-anti-TNFα and anti-TNFα groups, stratified by variant. Values were calculated using ELISA O.D. results, by dividing V4/V3 value, only for vaccinees with samples for both time points. (**A**–**C**) Statistical analysis was carried out using independent sample Kruskal–Wallis test. *—*p* < 0.0332, **—*p* < 0.0021, ***—*p*< 0.0002, ****—*p* < 0.0001.

The original Wuhan-Hu-1 virus was completely replaced by viral variants [32,33]. We, therefore, examined serologic responses against SARS-CoV-2 VOCs beta, gamma, delta and omicron BA.1 with ELISA. Sera binding to the Wuhan-Hu-1 strain RBD was correlative to anti-S levels (Supplementary Figure S3). In agreement with previous reports [34,35], while the sera of vaccinated subjects cross-reacted to some degree with all VOCs, there was an overall reduced reactivity against the omicron VOCs, followed by beta, gamma and delta (Figure 6A). This was kept for all groups (HCs, non-anti-TNFα and anti-TNFα) (Supplementary Figure S4), with the anti-TNFα group exhibiting the lowest reactivity to every VOC tested (Figure 6B). This group also demonstrated the greatest relative decrease in binding against all RBDs over time, including the original Wuhan-Hu-1 strain and the three VOCs (Figure 6C), compared to the HC and non-anti-TNFα groups (*p* < 0.0001 and *p* < 0.0008, respectively).

The most significant difference between the groups was in binding to the beta VOC. To assess whether the anti-TNFα treatment affected antibody cross-reactivity regardless of the reduced antibody titers in this group, we calculated the “beta cross-reactivity score” for each visit three samples as the ratio between the O.D. values against the beta VOC and the Wuhan-Hu-1 strain. The anti-TNFα group showed an approximately 10% reduction in cross-reactivity toward the beta VOC, which was significantly lower compared to the HCs group (*p* = 0.0018), and an approximately 5% reduction compared to the IBD group (*p* = 0.0152) (Supplementary Figure S5A). Similar analyses for the gamma, delta and omicron VOCs did not yield differences between the groups (Supplementary Figure S5B,C). For visit four samples, the beta cross-reactivity score could be calculated only for 42% of the anti-TNFα group, due to an unmeasurable anti-beta response in most samples, demonstrating similar trends (Supplementary Figure S5D). Additionally, the cross-reactivity scores and O.D. values against the Wuhan-Hu-1 strain were correlated (r = 0.5601, *p* < 0.0001) (Supplementary Figure S6). In order to verify the underlying cause for the cross-reactivity reduction, we performed a multi-variate linear regression demonstrating that only O.D. values against the Wuhan-Hu-1 strain maintained a significant correlation with the cross-reactivity score (Supplementary Table S3). Hence, the anti-TNFα group had significantly lower cross-reactivity to the beta VOC due to the lower anti-RBD IgG titer characterizing this group, while the anti-TNFα treatment itself had no direct effect on the cross-reactivity.

### 3.4. Additional Predictors of Lower Vaccine Responses

In the univariate analysis, we noticed that, in addition to the anti-TNFα treatment, older age and a longer interval between the second vaccine dose and visit four were also associated with a lower serologic response at visit four (Supplementary Table S4). Those factors remained significant also in the multi-variate linear regression model (Supplementary Table S5).

### 3.5. The Vaccine Is Safe in Patients with IBD and Is Not Associated with IBD Exacerbation

The potential for long-term side effects, as well as IBD exacerbation, concerned patients and care givers. We evaluated IBD activity using clinical and laboratory variables. To this end, no serious adverse events (SAEs) were registered. IBD activity, which was comparable in patients treated or not treated with anti-TNFα after the first and second vaccine doses [12], remained similar at the six- compared to the one-month time points (Supplementary Figure S7).

## 4. Discussion

Anti-TNFα is a mainstay therapy in IBD treatment [2]; however, it may be associated with increased susceptibility to infections [2,4] and a lower vaccine response [5,6]. In light of the COVID-19 pandemic, patients with IBD were encouraged to vaccinate [36,37], despite their exclusion from phase three trials [7,8]. In a previous study, we reported that patients with IBD treated with anti-TNFα agents developed significantly lower antibody responses to the SARS-CoV-2 mRNA vaccine compared to those untreated with anti-TNFα or HC [12]. However, the durability and breadth of this response are unknown. In the current study, we aimed to investigate the effect of the TNFα blockade on longevity, cross-reactivity and B cell response of SARS-CoV-2 vaccine-induced responses.

Hereby, we showed that patients with IBD, regardless of treatment, and HCs had reduced anti-S levels six months after two doses of the BNT162b2 vaccine. Anti-S levels decreased at a greater rate in patients with IBD treated with anti-TNFα, further increasing the gap between these patients to those receiving other therapies, or HCs. Importantly, the only seronegative subjects at six months were patients with IBD, mostly treated with anti-TNFα. Our results were consistent with recent reports focusing on HCs, showing that antibodies were waning gradually in a two-phase decay scheme, reaching a 10–20-fold reduction after six months [23,24]. A study in patients with IBD [38] recently reported that biological therapy (including anti-TNFα) in patients vaccinated with two doses of BNT162b2 (Pfizer) led to reduced anti-S levels compared to HCs vaccinated with two doses of the mRNA 1273 (Moderna). In our study, participants were vaccinated with the same vaccine regiment. Moreover, we provided a comprehensive analysis of immunologic response to the vaccination, including serological activity, seroconversion and B cell response. Longitudinal studies in patients with immune-mediated inflammatory disease or chronic inflammatory diseases (CIDs) treated with anti-TNFα agents, reported that anti-S levels five to six months post vaccination were significantly decreased compared to HCs or patients with CID not treated with anti-TNFα [39,40,41], further supporting the relevance of our data beyond IBD. The lower anti-S levels at the one-month time point may be explained by the importance of TNFα for B cell stimulation [42,43] and proliferation [44], while the faster decrease in titers points to the importance of TNFα for plasma cell survival, as previously described [45]. The TNFα blockade has been associated with reduced antibody responses to other vaccines, such as influenza and pneumococcal [5,46]. Specifically, B cell responses were reduced in response to the anti-TNFα treatment in patients with rheumatoid arthritis, following the seasonal influenza vaccination [47]. In contrast to the decreased B cell responses by the TNFa blockade, T cell activity was unaffected [38,48,49].

Additionally, the correlation between the anti-S levels and neutralization or inhibition activities demonstrated at the one-month post vaccination time point [12] remained at six months post vaccination. Correspondingly, all subjects had reduced neutralization and inhibition capabilities compared to the one-month time point, with a steeper decay in patients treated with anti-TNFα. Furthermore, there was a discordant correlation between inhibitory activity and anti-S levels or neutralizing activity six months after vaccination. The inhibition of RBD:ACE2 binding by antibodies is considered important for preventing SARS-CoV-2 infection. Yet, recently, it became clear that other targets may also be important in providing protection, such as inhibiting conformational rearrangements within the spike protein or preventing membrane fusion, and, thus, viral entry [50,51]. Therefore, the discordant between the pseudo-viral assay and the inhibitory ELISA may be explained by the fact that the first assay was affected by multiple antibodies inhibiting viral entry, while the latter was only affected by antibodies blocking the RBD:ACE2 interaction.

Several studies addressed the memory response in healthy and convalescent vaccinated individuals, showing that anti-SARS-CoV-2 memory B cell frequencies continued to increase [23,52]. In contrast, we hereby showed, for the first time, that patients with IBD treated with anti-TNFα demonstrated a reduction in RBD-specific B cells over time, suggesting that the TNFα blockade may also interfere with memory B cell differentiation and survival. This is crucial, since memory B cells are specifically important for eliciting a fast and effective response upon reinfection [30]. Notably, patients with IBD treated with anti-TNFα did not elicit severe outcomes to infections [53,54], implying that not only B cell responses participate in the protection against SARS-CoV-2 [55,56,57].

Anti-RBD titers correlated with cross-reactivity to VOCs, meaning that lower anti-S levels were accompanied by lower fractions of cross-reactive antibodies. Interestingly, cross-reactivity to the omicron VOC decreased dramatically compared to all other VOCs, as reported elsewhere [58,59,60]. Patients treated with anti-TNFα had lower anti-S levels and, thus, had lower cross-reactivity to VOCs. While vaccine effectiveness against infections was reported to decrease from 88% to 47% over the half-year elapsing from vaccination in HCs [61], the combination of lower sera function and lower cross-reactivity may suggest that patients treated with anti-TNFα are at high risk for breakthrough infections. This concern was unsupported by retrospective data from Israel focusing on the infection rate during the first three months after vaccination, without a specific increase in patients with IBD [62]. Furthermore, only two patients in our cohort experienced breakthrough infection over the six months, one treated with adalimumab and seronegative, and one treated with 6-mercaptopurine, having high anti-S levels. Thus, additional modifiers of breakthrough infections and their severity may be relevant.

More importantly, besides the anti-TNFα treatment, older age was an independent predictor of a lower serologic response (Supplementary Figure S8). Older age is a risk factor for severe COVID-19 [53,63] and poor vaccine effectiveness [12,64]. Thus, specific attention to vaccinating older patients who are also treated with anti-TNFα is warranted. Interestingly, a recent study focusing on patients >60 years compared to those aged 20–44 demonstrated significantly decreased responses to BNT162b2, and their resurrection after a third vaccine dose [65]. Another independent factor of a lower serologic response was the time interval from the second vaccine dose (Supplementary Figure S9). Subjects were assessed at a median of 176 days post vaccination. Anti-S levels were reported to decline in HCs four and six months post vaccination. It was shown that six months post vaccination, 16.1% of participants became seronegative [66,67]. In our cohort, none of the HCs became seronegative, while 17% of the patients with IBD treated with anti-TNFα became seronegative. These data further support the need for patients treated with anti-TNFα agents, specifically older ones, to receive an early booster vaccine.

Since the introduction of SARS-CoV-2 vaccines, concerns regarding their long-term safety were repeatedly raised [68,69]. In patients with immune-mediated disorders, concerns also included disease exacerbation [70,71,72,73]. Reassuringly, no IBD exacerbation was noticed in our patients—either clinical activity or inflammatory indices (Supplementary Figure S6). Furthermore, no SAEs (i.e., death or hospitalization) were reported. AEs were similar in all subjects and were mostly in the first month following vaccination, as reported previously [12]. No IBD exacerbation was observed, regardless of disease activity at baseline. Our prospective data were supported by health maintenance organization data from Israel reporting similar exacerbation rates between vaccinated and unvaccinated patients with IBD [74].

Our study was a prospective Israeli multi-center study comprehensively investigating multiple aspects of BNT162b2 vaccine serologic responses in patients with IBD [12]. By longitudinally evaluating the dynamics of immune responses, this was the first study prospectively and comprehensively addressing long-term vaccine responses in patients with IBD stratified according to therapy, specifically antibody responses, including anti-S levels, neutralizing and inhibitory activity, circulating B cell responses as well as responses to VOCs. Additional advantages included the high patient persistence rate, where most patients recruited before the first vaccine dose, remaining at follow up until the 6-month time point. Importantly, in addition to following 57 patients with IBD treated with anti-TNFα, we also followed 94 patients with IBD untreated with anti-TNFα, whether treated with other IBD therapies or untreated. Thus, we were able to report long-term vaccine responses in these sub-groups, as well as detecting disease activity through clinical scores and inflammatory indices, and we were able to provide prospective data reassuring data regarding the lack of SAEs and IBD exacerbation (Supplementary Figure S7). The relatively young age of participants was expected in patients with IBD and added to the applicability and importance of our data.

We acknowledge the limitations mainly related to the baseline cohort presented in our previous report [12]. These included small numbers of patients treated with steroids and immunomodulators, a limitation relevant to most other reports in IBD focusing mainly on anti-TNFα therapies, assumed to highly affect vaccine responses [13,15,75,76]. Additional limitations included differences in gender ratio in the IBD and HCs groups at baseline and the use of only one vaccine type.

To conclude, our study provided prospective, controlled evidence for long-term anti-TNFα dependent impairment in BNT162b2 vaccine responses, alongside evidence for its safety in patients with IBD. Justification for boosting approaches in this population was suggested [77]. Taking together our data regarding the combination of decreased immune responses and decreased cross-reactivity in patients with IBD treated with anti-TNFα with recent studies suggesting that immunosuppressed individuals might contribute to the emergence of new SARS-CoV-2 variants [78], this population should be monitored closely and addressed accordingly.

## Figures and Tables

**Table 1:** Demographic characteristics of participants.

Characteristics	Anti-TNFα N = 57	Non-Anti-TNFα N = 94	HC N = 89	*p* Value
Mean age, years (SD)	38.2 (14.1)	39.3 (13.4)	38.9 (12.2)	0.888
Female, n (%)	20 (35.1)	40 (42.6)	62 (69.7)	**<0.001**
Origin, n (%)				
Ashkenazi	28 (49.1)	42 (44.7)	48 (53.9)	0.457
Non-Ashkenazi	29 (50.9)	52 (55.3)	41 (46.1)	
Mean BMI, kg/m^2^ (SD)	25.5 (4.1)	24.3 (4.5)	25.1 (5.3)	0.329
Smoking status, n (%)				
Present	4 (7.0)	8 (8.5)	8 (9.0)	0.140
Past	4 (7.0)	7 (7.4)	0 (0)	
No	49 (86.0)	79 (84.0)	81 (91.0)	
Comorbidities ^a^, n (%)	5 (8.8)	6 (6.4)	5 (5.6)	
IBD phenotype, n (%)				
CD	47 (82.5)	50 (53.2)	-----	**<0.001**
UC	7 (12.3)	36 (38.3)	-----	**0.001**
IPAA	2 (3.5)	5 (5.3)	-----	
IBD-unclassified	1 (1.8)	3 (3.2)	-----	
Disease activity ^b^, n (%)				
Remission	42 (75.0)	54 (60.0)	-----	0.074
Active	14 (25.0)	36 (40.0)	-----	
Current medication, n (%)				
Infliximab	29 (50.9)	-----	-----	
Adalimumab	26 (45.6)	-----	-----	
Vedolizumab	-----	23 (24.5)	-----	
Ustekinumab	-----	8 (8.5)		
5-ASA	5 (8.8)	33 (35.1)	-----	
Steroids	1 (1.8)	7 (7.4)	-----	
Immunomodulators ^c^	8 (14.0)	4 (4.3)	-----	
JAK inhibitor	-----	5 (5.3)		
No medical treatment	-----	29 (30.9)	-----	

^a^ Comorbidities were present in 21 patients overall and included mainly asthma (6), diabetes (5), high blood pressure (5) and celiac (2). The rest were fatty liver disease, hypothyroidism, ankylosing spondylitis and prostate cancer. ^b^ Disease activity was quantified clinically with validated questionnaires. ^c^ Including 6-mercatopurine, azathioprine and methotrexate. Abbreviations: HC—healthy controls; BMI—body mass index; CD—Crohn’s disease; UC—ulcerative colitis; IBD-unclassified; IPAA—ileal pouch–anal anastomosis; 5-ASA—5-aminosalicylic acid; JAK—Janus kinase.

## Data Availability

The data underlying this article are available in the article and in its online Supplementary Materials.

## Supplementary Materials

The following supporting information can be downloaded at: https://www.mdpi.com/article/10.3390/vaccines10081186/s1, Supplementary Table S1: Geometric mean concentrations (GMCs) of IgG against the S-antigen of the 3 groups at each visit. Supplementary Table S2: Demographic characteristics of participants donating PBMCs. Supplementary Table S3: Factors associated with beta cross-reactivity score (multi-variate linear regression). Supplementary Table S4: Factors associated with serologic response (univariate analysis). Supplementary Table S5: Factors associated with serologic response (multi-variate linear regression). Supplementary Figure S1: (A) Serologic and (B) inhibition responses to sub-groups within non-anti-TNFα group stratified according to their medical treatment: no medical treatment (31, in black), 5-ASA (18, in dark grey), vedolizumab (22, in light grey), other medical treatments with relatively small sub-groups (steroids-4, immunomodulators-5, ustekinumab-7, tofacitinib-5 and clinical study drug-2 in brown). Black solid lines denote median, dashed lines denote IQR 25-75. Similar outcomes were obtained in neutralization assays (data not shown). Supplementary Figure S2: Immunoglobulin levels and lymphocytes sub-populations among patients with IBD were stable at all 4 visits. Supplementary Figure S3: Correlation between ELISA results against Wuhan-1 RBD and anti-S level. Supplementary Figure S4: Binding of vaccinees sera to RBD from Wuhan-1 strain and variants of concern. Ability of healthy controls (HCs), patients with IBD receiving non-TNFα treatment (non-anti-TNFα) and patients with IBD receiving non-TNFα treatment (non-anti-TNFα) sera to bind SARS-CoV-2 RBD. RBD was from Wuhan-1 strain and variants of concern (VOCs)—beta, gamma, delta and omicron (Wuhan-1 in orange, beta in light blue, gamma in light grey, delta in dark grey and omicron in black). Binding was measured with ELISA for two time points—1 month post vaccination (visit 3, filed circles) and 6 months post vaccination (visit 4, open triangles). Dotted line indicates mean O.D. value from 5 sera samples before vaccination. Statistical analysis was carried out using independent sample Kruskal–Wallis test. *—*p* < 0.0332, **—*p* < 0.0021, ***—*p* < 0.0002, ****—*p* < 0.0001. Supplementary Figure S5: Cross-reactivity score to VOCs at V3. Cross-reactivity scores, calculated by deviation of. (A) beta O.D. results by Wuhan-1 strain O.D. results. (B) Gamma O.D. results by Wuhan-1 strain O.D. results. (C) Delta O.D. results by Wuhan-1 strain O.D. results. (D) Omicron O.D. results by Wuhan-1 strain O.D. results. Separated to HC (purple), non-anti-TNFα (blue) and anti-TNFα (red) groups. Statistical analysis was carried out using independent sample Kruskal–Wallis test *—*p* < 0.0332, **—*p* < 0.0021. Supplementary Figure S6: Correlation between beta cross-reactivity score and the O.D. values against Wuhan-1 strain, calculated with spearman correlation, r = 0.5601, *p*-value < 0.0001. Dots are colored by groups (HC in purple, non-anti-TNFα in blue and anti-TNFα in red). Supplementary Figure S7: Disease activity during follow up. Activity was measured with validated questionnaires. Bars represent the average score of either HBI for CD or SCCAI for UC, stratified according to treatment (with and without anti-TNFα), after 1- and 6-month vaccine doses (visit 3, black; visit 4, grey). Error bars denote SD. The difference between the groups was not significant using independent sample Kruskal–Wallis test. Supplementary Figure S8: Correlation between age and serologic response. Supplementary Figure S9: Correlation between time interval from second vaccine dose and serologic response.
