# Supplementary material for: Anti-TNFα Treatment Impairs Long-Term Immune Responses to COVID-19 mRNA Vaccine in Patients with Inflammatory Bowel Diseases
Source: Vaccines (Basel). 2022 Jul 26;10(8):1186. doi: 10.3390/vaccines10081186 (PMC9330864; doi:10.3390/vaccines10081186)
Supplement: Supplementary file 1 [file vaccines-10-01186-s001.zip › vaccines-1801080-supplementary.pdf]

Supplementary Materials:

Supplementary Table S1: Geometric Mean Concentrations (GMCs) of IgG against the S-antigen of the 3 groups at each visit

Visit No	Group	GMC	95% Confidence Interval for GMC		ANOVA		
			Lower Bound	Upper Bound	*P* value HC	*P* value Non-anti-TNFα	*P* value Anti-TNFα
3	HC	10979.3	9396.0	12829.5	-----	.272	**<.001***
	Non-TNFα	8320.4	6630.0	10441.6	.272	-----	**<.001***
	TNFα	3787.0	2732.0	5249.3	**<.001***	**<.001***	-----
4	HC	1253	1023	1534	-----	.106	**<.001***
	Non-TNFα	830	632	1090	.106	-----	**<.001***
	TNFα	187	122	288	**<.001***	**<.001***	-----

This table emphasizes the significant difference in serologic response between anti-TNFα and non-anti-TNFα and HC after first and second vaccine doses.
*Testing differences between groups with Bonferroni correction for multiple comparisons
Abbreviations: HC=healthy controls.

Supplementary Table S2: demographic characteristics of participants donating PBMCs

Characteristic	Anti-TNFα N=4	Non-anti-TNFα N=4	HC N=5	*P* value
Mean age, years (SD)	43.83 (10.81)	32.15 (6.133)	31.06 (8.097)	0.0774*
Current medication, n (%)				
Anti-TNFα	4 (100)	-----	-----	
Vedolizumab	-----	2 (50)	-----	
5-ASA	-----	0 (0)	-----	
Other	-----	1 (25)	-----	
No medical treatment	------	1 (25)	-----	

Abbreviations: HC=healthy controls, 5-ASA= 5-aminosalicylic acid
*Testing differences between groups with independent-samples Kruskal-Wallis test

Supplementary Table S3: Factors associated with beta cross-reactivity score (multivariate linear regression)

Variable		B (95% CI)	P value
Treatment	Anti-TNFα	-.088	.532
	Non-anti-TNFα	.034	.118
	HC	Reference	

Whuan-1 O.D.	.168 (.134-.201)	**<.001**

Supplementary Table S4: Factors associated with serologic response (univariate analysis)

Variable		n/N	GMC (95%CI)/Spearman's rho	p-value
Treatment group	HC	61/181	1253 (1023-1534)	**<0.001**
	Non-anti-TNFα	77/181	830 (632-1090)	
	Anti-TNFα	43/181	187 (122-288)	
Treatment in non-anti-TNFα	Vedolizumab	21/77	1270 (726-2221)	0.279
	5ASA	19/77	788 (404-1535)	
	No treatment	22/77	669 (463-966)	
	other	15/77	671 (321-1401)	
Gender	Male	83/181	567 (413-779)	0.128
	Female	98/181	770 (601-987)	
Age (years)		178/181	Rho=-0.355	**<0.001**
BMI (Kg/m^2)		178/181	Rho=-0.067	0.376
Diagnosis	CD	73/120	384 (273-540)	0.090
	UC	39/120	780 (503-1210)	
	IBDU	3/120	626 (23-16518)	
	IPAA	5/120	337 (41-2771)	
HBI score	Remission	45/69	350 (222-552	0.505
	Active	24/69	452 (243-841)	
SCCAI score	Remission	28/42	725 (443-1187)	0.697
	Active	14/42	862 (363-2050)	
Hemoglobin levels (g/dL)		164/181	Rho=-0.075	0.341
WBC levels (K/micL)		165/181	Rho−0.047	0.547
CRP levels (mg/dL)		163/181	Rho=-0.073	0.356
Serology visit3/visit2		170/181	Rho=-0.184	**0.017**
Δ 2nd vaccine dose &visit 4		181/181	Rho=-0.207	**0.005**
Inhibition ELISA		180/181	Rho=0.208	**0.005**
Pseudovirus inhibition		181/181	Rho=0.661	**<0.001**

Supplementary Table S5: Factors associated with serologic response (multivariate linear regression)

		B* (95% CI)	*P* value
Treatment	Anti-TNFα	-2.1 (-2.5 to -1.7)	<0.001
	Non-anti-TNFα	-0.6 (-0.9 to -0.2)	0.002
	HC	reference	
Age (years)		-0.03 (-0.04 to -0.02)	<0.001
Difference between Vac2 & visit 4 (days)		-0.01 (-0.02 to -0.01)	<0.001

*Standardized Beta coefficients were obtained from linear regression. Abbreviations: HC=healthy controls.

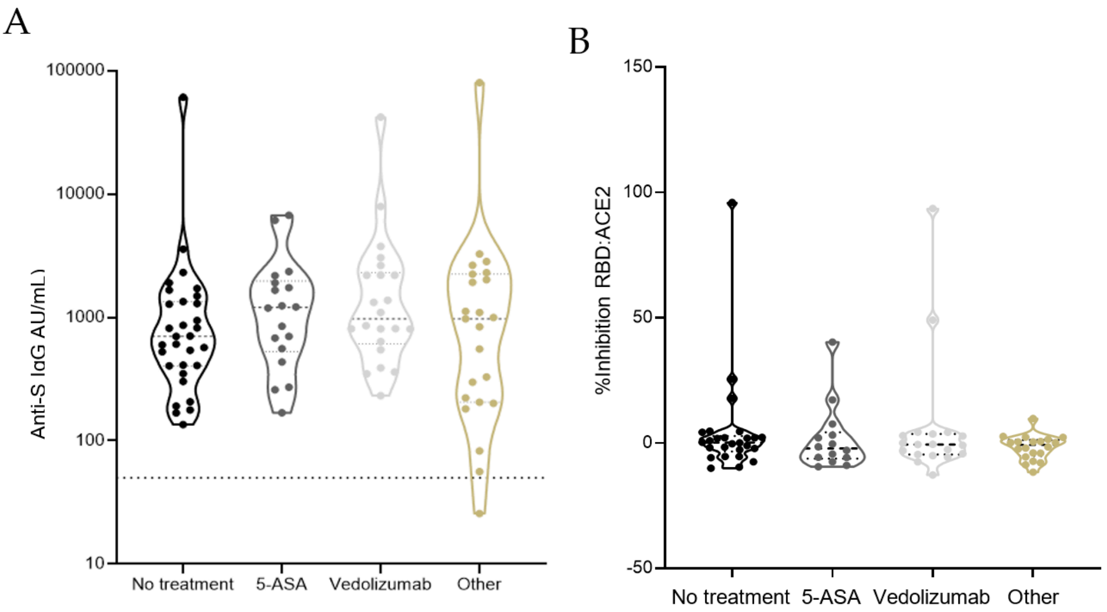

Supplementary Figure S1: (A) Serologic and (B) inhibition responses to subgroups within non-anti-TNFα group stratified according to their medical treatment: no medical treatment (31, in black), 5-ASA (18, in dark grey), Vedolizumab (22, in light grey), other medical treatment which relatively small subgroups (Steroids-4, immunomodulators-5, ustekinumab-7, tofacitinib-5 and clinical study drug-2 in brown). Black solid lines denote median, dashed lines denote IQR 25-75. Similar outcomes were obtained in neutralization assays (data not shown).

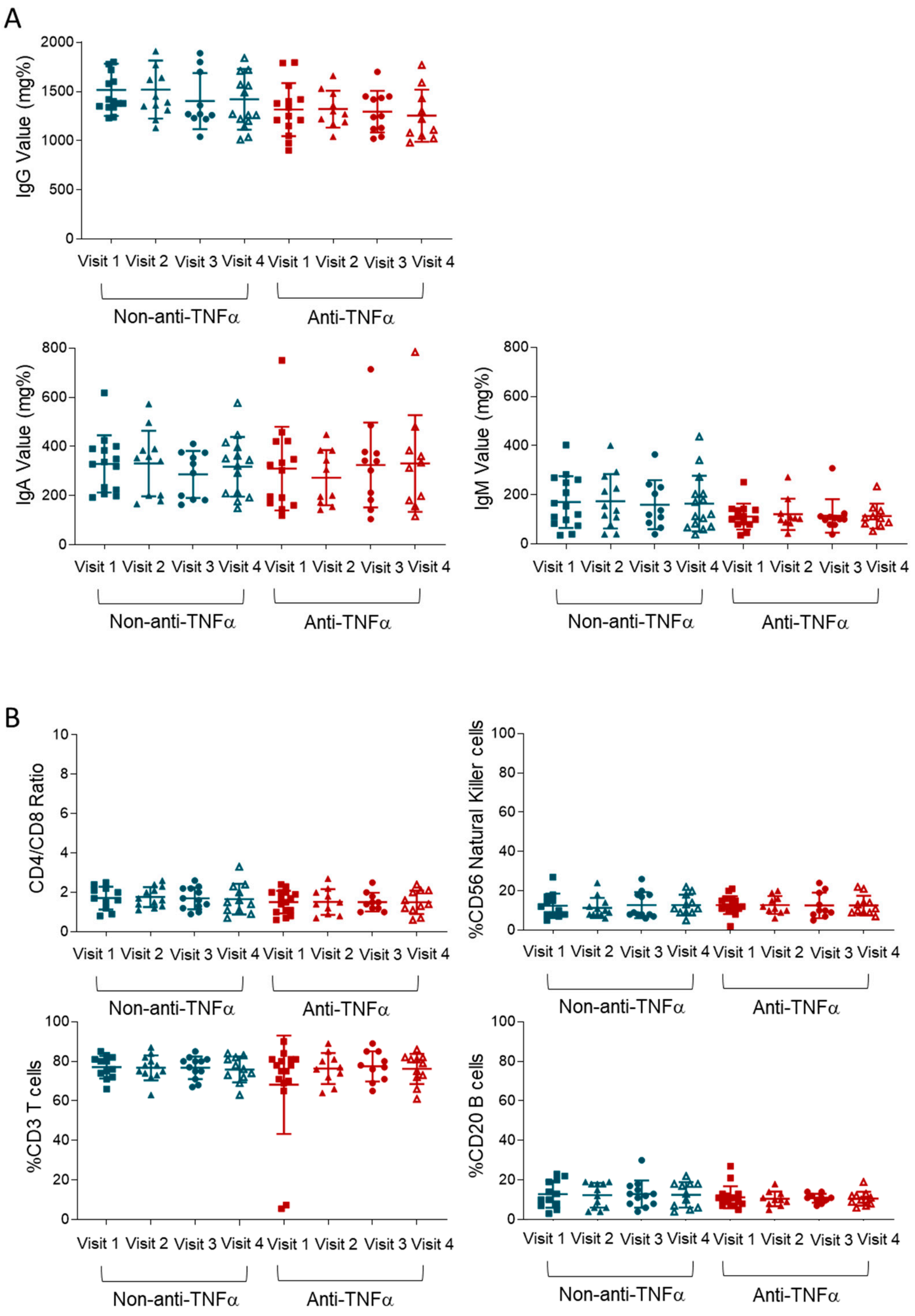

Supplementary Figure S2: Immunoglobulin levels and lymphocytes subpopulations among patients with IBD are stable at all 4 visits. (A) IgG, IgA and IgM levels (mg%) (B) Percentages of T (CD3 positive), B (CD20 positive) and NK (CD56 positive) cells among leukocytes as well as the ratio between CD4/CD8 T cells. Both tested in patients with IBD treated (in red) or not with anti TNFα (in blue) in all 4 visits (visit 1 – filled squares, visit 2 – file triangles, visit 3 – filled circles, visit 4 – empty triangles).

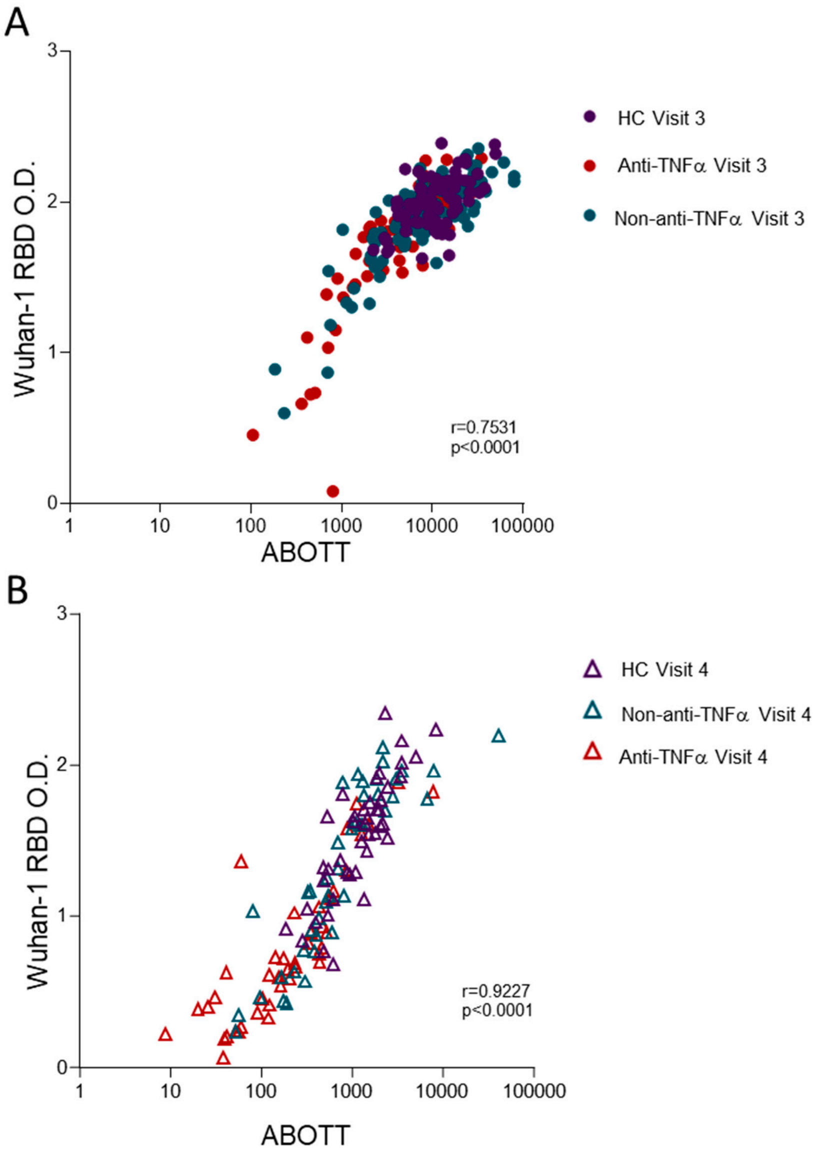

Supplementary Figure S3: Correlation between ELISA results against Wuhan-1 RBD and anti-S level. Each dot represents one sample, the Y axis indicating the Wuhan-1 strain ELISA results while the X axis indicating ABBOTT kit results. HC in purple, Non-anti-TNFα in blue and anti-TNFα in red. (A) results from visit 3, filled circles. Correlation calculated with Spearman correlation, r = 0.7531, p-value < 0.0001. (B) visit 4, open triangles. Correlation calculated with Spearman correlation, r = 0.9227, p-value < 0.0001.

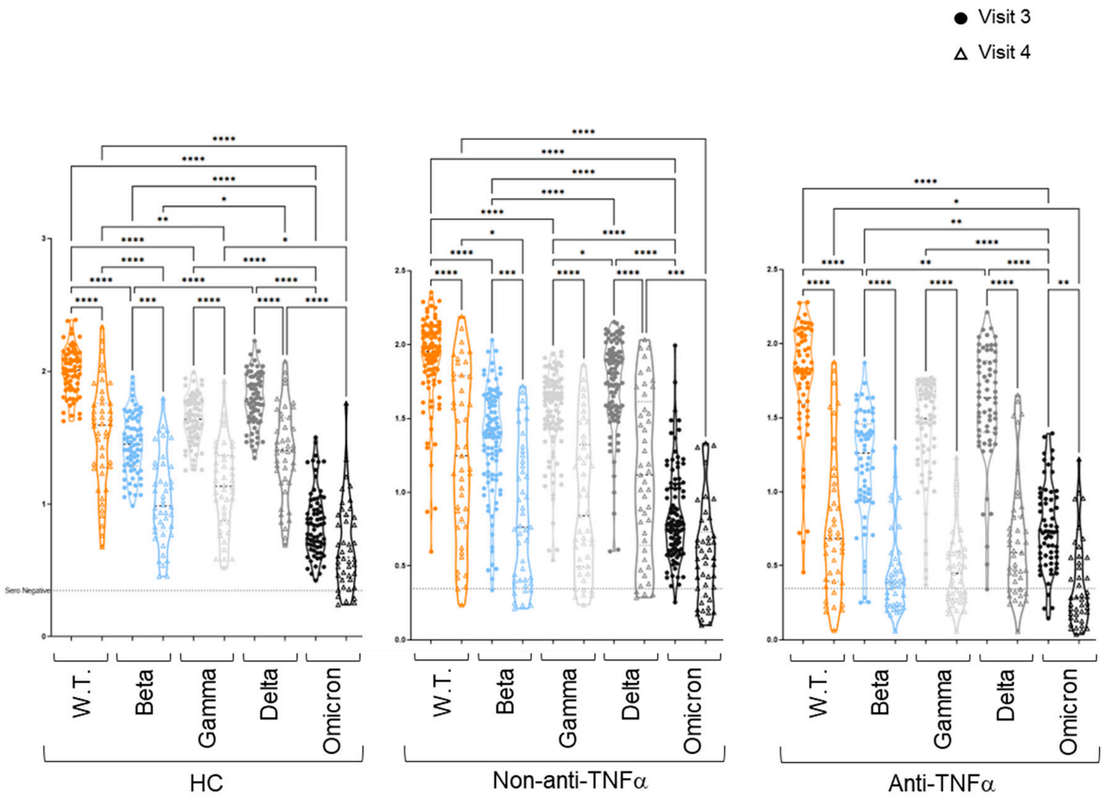

Supplementary **Figure S4: Binding of vaccinees sera to RBD from Wuhan-1 strain and variants of concern**. Ability of healthy controls (HC), patients with IBD receiving non-TNF*α* treatment (Non-anti-TNF*α*), and patients with IBD receiving non-TNF*α* treatment (Non-anti-TNF*α*) sera to bind SARS-CoV-2 RBD. RBD is from Wuhan-1 strain and variants of concern (VOCs)– Beta, Gamma, Delta and Omicron (Wuhan-1 in orange, Beta in light blue, Gamma in light grey, Delta in dark grey and Omicron in black). Binding is measured by ELISA, for two time points – 1-month post vaccination (Visit 3, filed circles) and 6-month post vaccination (Visit 4, open triangles). Dotted line indicating mean O.D. value from 5 sera samples before vaccination. Statistical analysis was carried out using independent-samples Kruskal-Wallis test * p<0.0332, ** p<0.0021, *** p< 0.0002, **** p<0.0001.

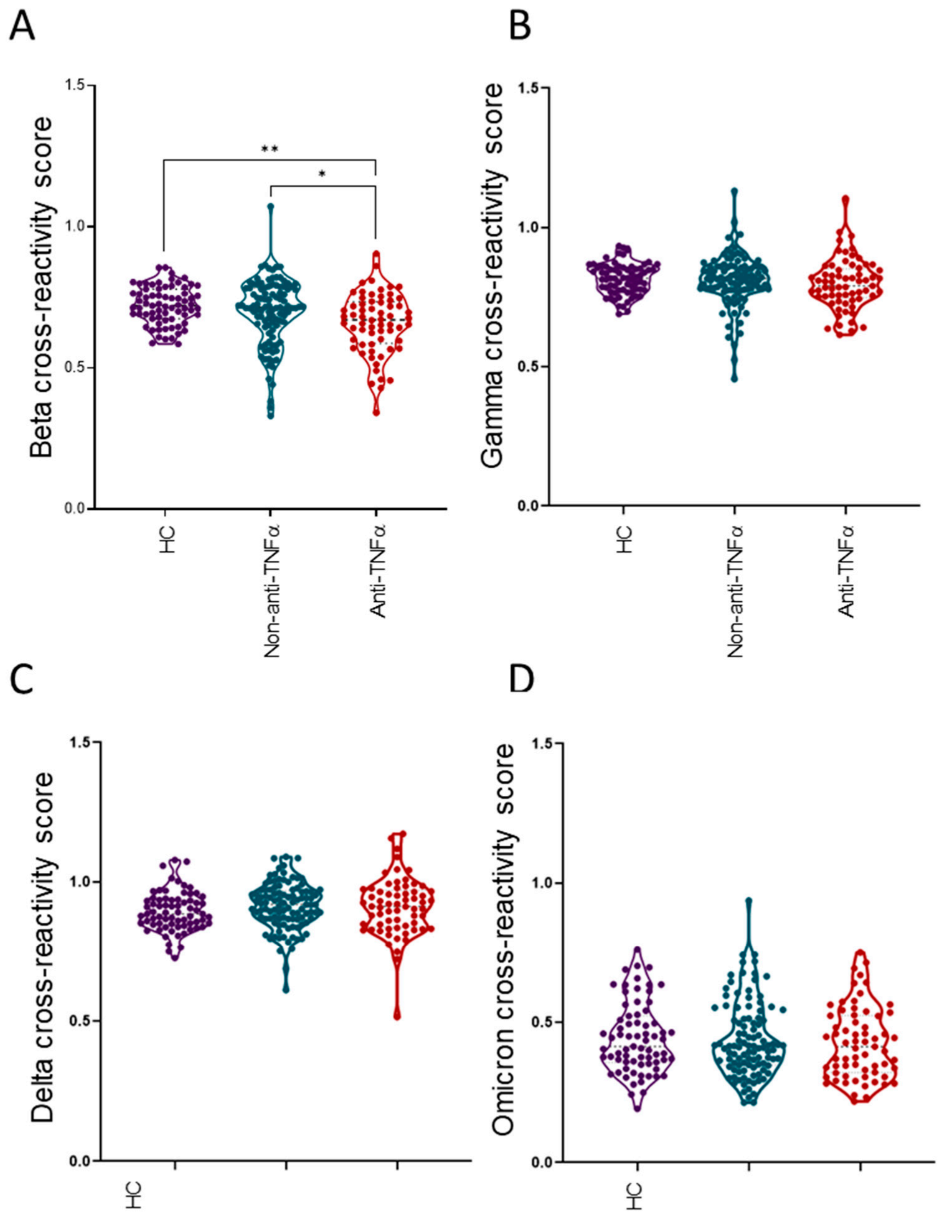

Supplementary Figure S5: Cross-reactivity score to VOCs at V3. Cross reactivity scores, calculated by deviation of (A) Beta O.D. results by Wuhan-1 strain O.D. results (B) Gamma O.D. results by Wuhan-1 strain O.D. results (C) Delta O.D. results by Wuhan-1 strain O.D. results, (D) Omicron O.D. results by Wuhan-1 strain O.D. results. Separated to HC (purple), Non-anti-TNF*α* (blue), and anti-TNF*α* (red) groups. Statistical analysis was carried out using independent-samples Kruskal-Wallis test * - p<0.0332, ** - p<0.0021.

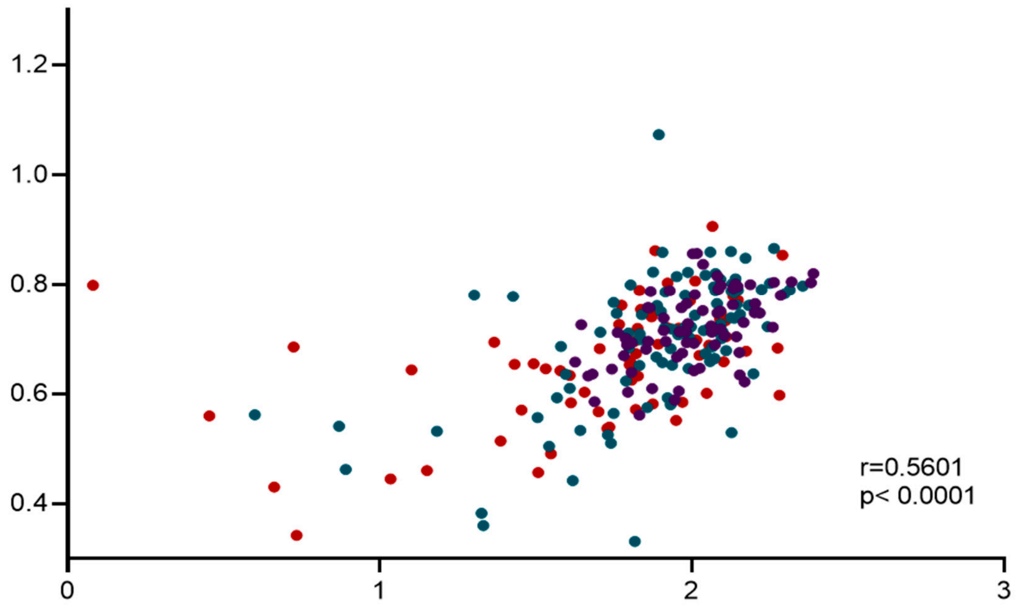

Supplementary Figure S6: Correlation between beta cross-reactivity score and the O.D. values against Wuhan-1 strain, calculated with spearman correlation, r = 0.5601, p-value < 0.0001. Dots are colored by groups (HC in purple, non-anti-TNFα in blue and anti-TNFα in red).

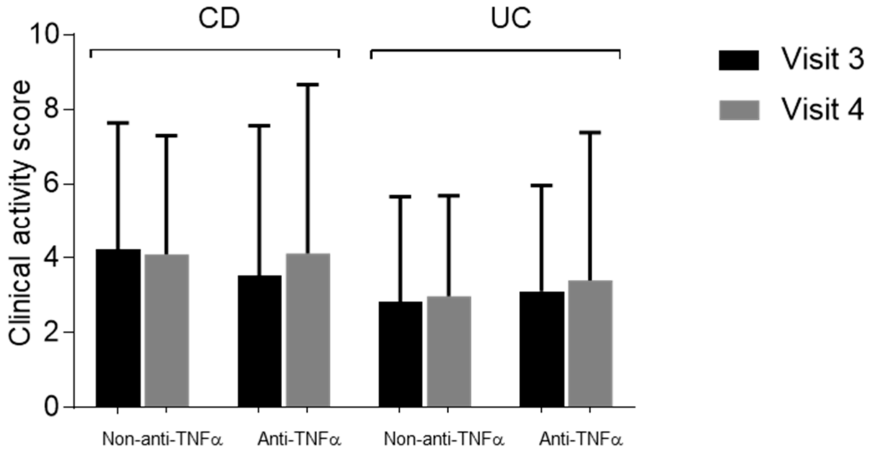

Supplementary Figure S7: Disease activity during follow up. Activity was measured by validated questionnaires. Bars represent the average score of either HBI for CD or SCCAI for UC, stratified according to treatment (with and without anti-TNFα), after 1- and 6- months two vaccine doses (visit 3, black; visit 4, grey, respectively). Error bars denote SD. The difference between the groups was not significant using Independent-Samples Kruskal-Wallis Test.

Abbreviations: HBI=Harvey-Bradshaw Index; SCCAI=Simple Clinical Colitis Activity Index, UC=Ulcerative colitis, CD=Crohn's disease

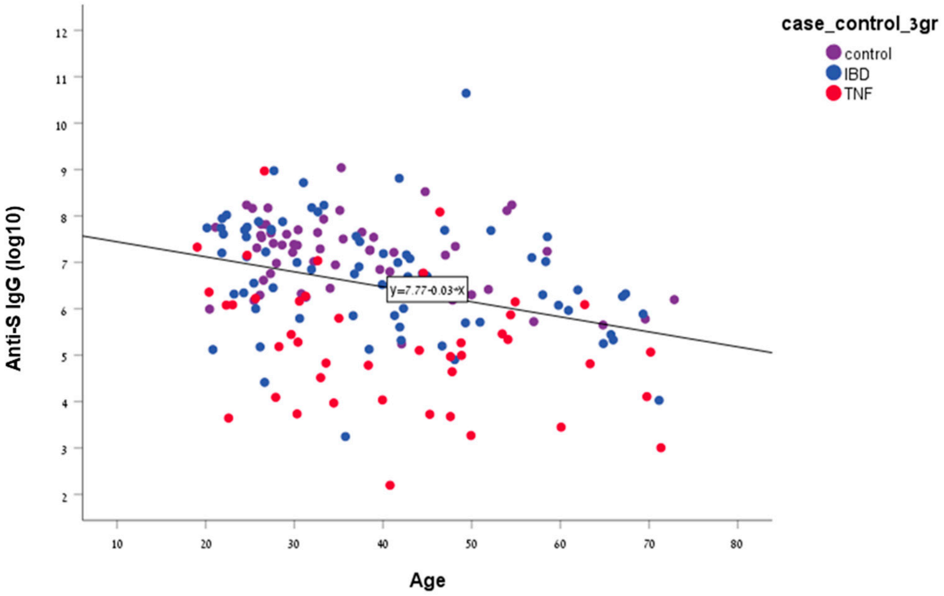

Supplementary Figure S8: Correlation between age and serologic response. Correlation between older age and lower levels of IgG anti-S antibodies in the three study groups six months after second vaccine doses. Anti-TNFα in red, non-anti-TNFα in blue, HC in purple circles.

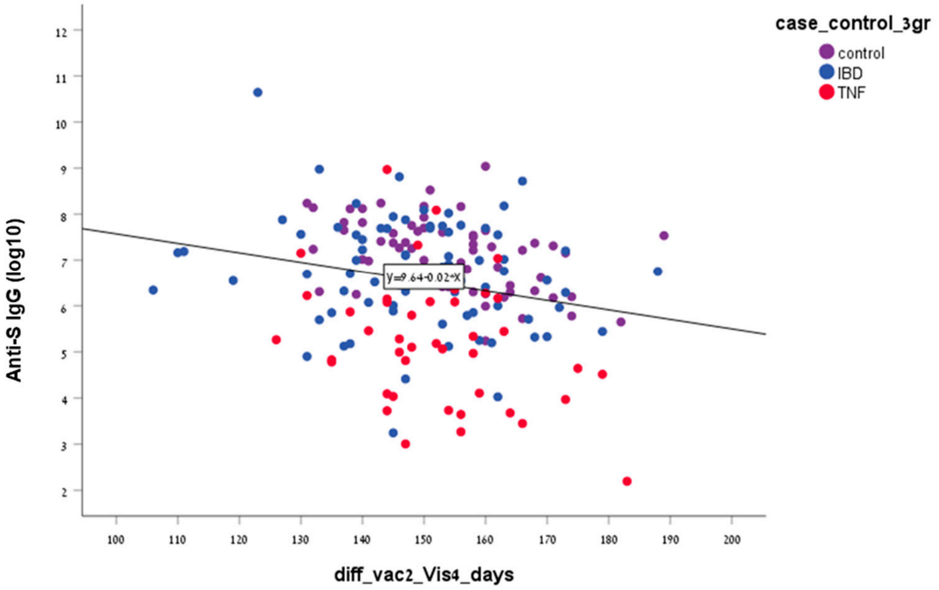

Supplementary Figure S9: Correlation between time interval from second vaccine dose and serologic response. Correlation between time after second vaccine dose and IgG anti-S antibodies in the three study groups six months after second vaccine doses. Anti-TNFα in red, non-anti-TNFα in blue, HC in purple circles.
